# Implementation of diagnostic resources for cancer in developing countries: a focus on PET/CT

**DOI:** 10.3332/ecancer.2019.ed87

**Published:** 2019-01-31

**Authors:** Haydeé C Verduzco-Aguirre, Gilberto Lopes, Enrique Soto-Perez-De-Celis

**Affiliations:** 1 Department of Oncology, Instituto Nacional de Ciencias Médicas y Nutrición Salvador Zubirán, Vasco de Quiroga 15, Sección XVI, Tlalpan, Mexico City, Mexico; 2 Sylvester Comprehensive Cancer Center. University of Miami Miller School of Medicine Miami, FL, 33136, USA.; 3 Department of Geriatrics, Instituto Nacional de Ciencias Médicas y Nutrición Salvador Zubirán, Vasco de Quiroga 15, Sección XVI, Tlalpan, Mexico City, Mexico.

**Keywords:** positron-emission tomography, computed tomography, diagnostic test approval, technology assessment, biomedical, developing countries, economic evaluation

## Abstract

Positron emission tomography in conjunction with computed tomography (PET/CT) is a relatively novel diagnostic tool which has been proven to be clinically useful in various neoplasms. Currently, only a handful of developing countries have PET/CT capabilities, and in those that do units are mostly located in large urban areas, which limits their availability. The implementation of PET/CT units in low-and-middle income countries is hampered by their high cost, the difficulties associated with their operation, and the limited availability of trained personnel. Furthermore, although the clinical appropriateness of PET/CT is well defined in many scenarios, little is known about its cost-effectiveness, particularly in settings with limited resources. Here, we provide a brief overview of the challenges associated with the implementation of PET/CT in resource-limited settings, including some examples of available data on its cost-effectiveness.

## Introduction

Using limited resources in a prudent and effective way is an essential component of healthcare system planning. This is true not only for the adoption of new medications or therapeutic techniques, but also of novel diagnostic tests, which may be expensive to implement and maintain. Therefore, incorporating a diagnostic test into cancer care requires an evaluation not only of its accuracy, but also of the feasibility of its implementation. 18F-fluorodeoxyglucose (18F-FDG) positron emission tomography (PET) in conjunction with computed tomography (PET/CT) is a relatively novel modality that can be utilized throughout the spectrum of cancer care, including diagnosis, staging, response evaluation, follow-up, and treatment planning [1]. However, the usefulness of PET/CT varies according to each scenario, and only those indications which have shown to improve diagnostic performance, influence clinical practice, and impact patient outcomes favorably can be considered appropriate (Table 1). Other indications showing improved diagnostic performance but lacking evidence regarding impact on outcomes (such as the staging of breast cancer, the staging of colorectal cancer, and the diagnosis of pancreatic cancer) are considered to be potentially appropriate [1]. Furthermore, high-quality economic analyses of PET/CT are scarce, particularly in low-and-middle income countries (LMIC), and doubts exist regarding whether countries lacking basic resources should invest in this diagnostic method [2].

## Implementation of PET/CT in resource-limited settings

Currently, only a few countries offer PET/CT to a significant proportion of their population. According to the World Health Organization’s Global Atlas of Medical Devices, only 3% of upper-middle income, and 4% of lower-middle income countries possesses at least one PET scanner per million people, compared to 29% of high-income countries. Furthermore, 95% of low income countries and 92% of lower-middle income countries don’t have an available PET/CT unit [3].

**Figure 1.**
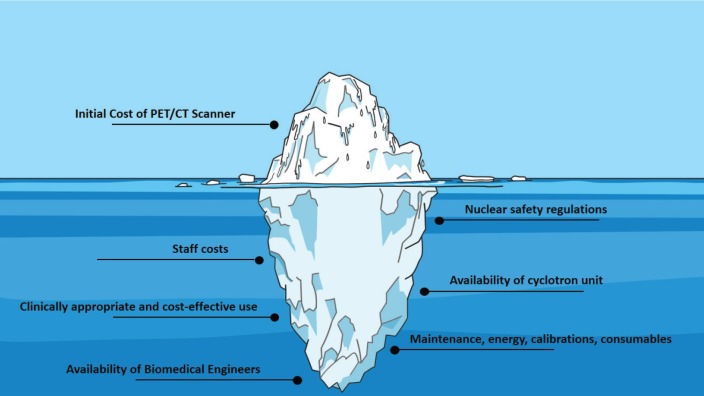
Challenges in the implementation and maintenance of a PET/CT unit.

Establishing a PET/CT center requires careful planning and an in-depth assessment of available resources. In fact, the initial cost of procuring the unit is just the tip of the iceberg, since this is followed by operational costs, added to the cost of human resources and staff (Figure 1) [4]. An analysis in a tertiary center in India, for example, found that the annual cost of a PET/CT unit was of $1,020,495, which consisted of 76% capital costs and 24% operating costs [5]. Furthermore, many countries lack personnel trained in the operation of a PET/CT unit, which represents a critical barrier to implementation. The global availability of biomedical engineers, for example, is widely variable, with many LMIC having less than one per million population [6].

Another barrier to the implementation of PET/CT is the need for positron-emitting radiotracers, which need to be produced using a cyclotron. These radiotracers have a short half-life and emit high energy radiation, which means that the cyclotron facility has to be located as near as possible to the scanner (four hours maximum), and that both facilities need to adhere to the highest safety standards [4, 7]. This means that new units usually require being concentrated within limited geographic areas. In fact, even in countries with available resources, nuclear medicine services are usually located in urban centers, leaving many regions without access to these technologies [3].

## Cost effectiveness of PET/CT

After a PET/CT unit is established, its appropriate clinical and financial utilization is of the utmost importance. Therefore, a fundamental step is the undertaking of cost-effectiveness analyses. A cost-effectiveness analysis compares the relative costs and outcomes of different procedures, with results presented as an incremental cost-effectiveness ratio (ICER), representing the average incremental cost for one additional unit of effect. Decision makers establish willingness-to-pay thresholds based on the ICER and other local measures, such as the per-capita gross domestic product, and use this information to support decisions at a national level [8]. The cost-effectiveness of PET/CT has been demonstrated in several clinical scenarios, with the strongest evidence coming from non-small-cell lung cancer (NSCLC) and head and neck squamous cell carcinoma (HNSCC) [2].

The use PET/CT for staging NSCLC has been shown to reduce the number of futile thoracotomies, with correct upstaging of 7% more patients when compared to conventional CT staging, sparing them from inappropriate surgery. In a Danish study, the number needed to treat (NNT) in terms of PET/CT scans in order to avoid a futile thoracotomy was 4.92, with an absolute risk reduction of futile thoracotomies of 20%. PET/CT was associated with an incremental cost of €3,927, resulting in an ICER of €19,314, which had a 90% probability to be cost effective with a willingness to pay threshold of €50,000 per avoided futile thoracotomy [9]. The avoidance of futile procedures is a relevant outcome, since this could not only spare patients from the morbidity of surgery, but also allow for a better selection of surgical candidates in areas with limited availability of specialized surgeons. That being said, current resource-stratified guidelines (which are based on the level of resources available in the practice area where care is being provided), such as those issued by the National Comprehensive Cancer Network (NCCN), do not include PET/CT for the evaluation of NSCLC, even in settings with enhanced resources, and instead recommend utilizing other tools such as conventional CT scanning and bone scans [10].

The cost-effectiveness of PET/CT in the evaluation of treatment responses in HNSCC was evaluated in the PET-Neck study, a UK-led non-inferiority trial comparing PET/CT-guided surveillance with planned neck dissection in patients who received chemoradiotherapy as primary treatment. With a median follow-up of 3 years, PET-Neck confirmed the non-inferiority of a PET/CT guided surveillance strategy, with patients in the surveillance group spared of neck dissection in about 80% of cases. Additionally, a cost-effectiveness analysis at a 2-year follow up reported a per-person cost saving of £1,492, and a gain of 0.08 quality-adjusted life years (QALYs) per person [11]. When long term outcomes were modelled, the average per-person lifetime saving was calculated at £1,485, with an additional gain of 0.13 QALYs, which was cost effective from the UK National Health Service (NHS) perspective [12].

In contrast, there are situations in which PET/CT has not reached acceptable ICER thresholds. In cervical cancer, for example, performing a PET/CT was significantly more costly and only minimally more effective than usual follow-up with other imaging modalities, with an ICER of over 1 million per QALY from the NHS perspective [13]. Therefore, it can be inferred that in countries where cervical cancer is more prevalent, the outcome probably would be unfavorable as well.

## Conclusions

Even though cost-effectiveness analyses support the use of PET/CT for certain scenarios in cancer care, the applicability of those results in LMICs is unknown. In centers and regions where PET/CT scanners have not yet been established, economic analyses should take into account implementation and maintenance cost, as well as the availability of tracers and of trained personnel. Although the WHO list of priority medical devices for cancer management includes PET/CT, many countries still lack other essential tools, such as CT scanners or radiotherapy facilities [3, 7]. Therefore, each country should undertake an in-depth assessment of specific needs, and prioritize those interventions which are more likely to improve patient outcomes and which have better cost-effectiveness ratios.

## Conflicts of Interest

The authors have no conflicts of interest to disclose.

## Funding

No funding was obtained for this manuscript.

## Figures and Tables

**Table 1.** PET/CT indications considered appropriate by the International Atomic Energy Agency (adapted from [1]).

Cancer Type	Indications			
	Diagnosis	Staging	Response assessment	Restaging/Suspected recurrence
Lung cancer	X	X		
Lymphoma		X	X	X
Melanoma		X		X
Ovarian cancer				X
Cervical cancer		X		X
Head and Neck Cancer	X		X	X
Colon cancer				X
Nasopharyngeal carcinoma		X	X	X
Gastrointestinal stromal tumors		X	X	X
Esophageal cancer		X		
Thyroid cancer				X
